# Machine-Learning-Based Tool to Predict Target Prostate Biopsy Outcomes: An Internal Validation Study

**DOI:** 10.3390/jcm12134358

**Published:** 2023-06-28

**Authors:** Enrico Checcucci, Samanta Rosati, Sabrina De Cillis, Noemi Giordano, Gabriele Volpi, Stefano Granato, Davide Zamengo, Paolo Verri, Daniele Amparore, Stefano De Luca, Matteo Manfredi, Cristian Fiori, Michele Di Dio, Gabriella Balestra, Francesco Porpiglia

**Affiliations:** 1 Department of Surgery, Candiolo Cancer Institute, FPO-IRCCS, Candiolo, 10060 Turin, Italy; volpi_gabriele@yahoo.it; 2 Department of Electronics and Telecommunications, Politecnico di Torino, 10129 Turin, Italy; samanta.rosati@polito.it (S.R.); noemi.giordano@polito.it (N.G.); gabriella.balestra@polito.it (G.B.); 3 Department of Oncology, Division of Urology, University of Turin, San Luigi Gonzaga Hospital, Orbassano, 10043 Turin, Italy; sabrinatitti.decillis@gmail.com (S.D.C.); stefano.granato91@gmail.com (S.G.); zamengo.davide@gmail.com (D.Z.); paoloverri05@gmail.com (P.V.); danieleamparore@hotmail.it (D.A.); delucastefano@yahoo.it (S.D.L.); matteo.manfredi85@gmail.com (M.M.); cristian.fiori@unito.it (C.F.); porpiglia@libero.it (F.P.); 4 Division of Urology, Department of Surgery, Annunziata Hospital, 87100 Cosenza, Italy; micheledidio@yahoo.it

**Keywords:** prostate cancer, artificial intelligence, prostate biopsy, machine learning

## Abstract

The aim of this study is to present a personalized predictive model (PPM) with a machine learning (ML) system that is able to identify and classify patients with suspected prostate cancer (PCa) following mpMRI. We extracted all the patients who underwent fusion biopsy (FB) from March 2014 to December 2019, while patients from August 2020 to April 2021 were included as a validation set. The proposed system was based on the following four ML methods: a fuzzy inference system (FIS), the support vector machine (SVM), k-nearest neighbors (KNN), and self-organizing maps (SOMs). Then, a system based on fuzzy logic (FL) + SVM was compared with logistic regression (LR) and standard diagnostic tools. A total of 1448 patients were included in the training set, while 181 patients were included in the validation set. The area under the curve (AUC) of the proposed FIS + SVM model was comparable with the LR model but outperformed the other diagnostic tools. The FIS + SVM model demonstrated the best performance, in terms of negative predictive value (NPV), on the training set (78.5%); moreover, it outperformed the LR in terms of specificity (92.1% vs. 83%). Considering the validation set, our model outperformed the other methods in terms of NPV (60.7%), sensitivity (90.8%), and accuracy (69.1%). In conclusion, we successfully developed and validated a PPM tool using the FIS + SVM model to calculate the probability of PCa prior to a prostate FB, avoiding useless ones in 15% of the cases.

## 1. Introduction

In the constant effort to promote patient-specific care, with the aim of diagnosing and treating different diseases, urologists are forced to manage large datasets and to interpret complex information [1]. Due to its potential application in accelerating and performing the analysis of this large and nonlinear amount of data, artificial intelligence (AI), has aroused great interest in the scientific community, leading to the development of specific algorithms that are able to consider different variables simultaneously [2].

AI and machine learning (ML) may represent the key technologies for performing reasoning, learning, and problem-solving tasks [3]. In the wide field of ML methods, fuzzy logic (FL) has proven to be a valuable tool in performing classification tasks in complex environments. Its main advantage is its capability in dealing with uncertainty, which is typical of biological systems and is often difficult to address in real life [4,5]. An important characteristic of FL is the possibility of obtaining an explanation of results. During the most recent decade, FL was often used in the medical field for the implementation of decision-support systems, with promising results [6,7].

Focusing on prostate cancer (PCa), the diagnostic accuracy of the single baseline characteristics of patients (e.g., PSA, digital rectal exploration (DRE), and multiparametric magnetic resonance imaging ([mp-MRI)) has proven to be suboptimal, opening the door to new approaches that are able to analyze all of these variables simultaneously [8].

In a previous work, we explored the potential of FL in the prediction of biopsy outcomes. The goal of our system was to identify the ideal candidate for fusion target biopsy (FB) [9]. The results were encouraging, but the system was not able to classify all patients.

The aim of the present work is to provide a system that is able to classify all patients. 

The system’s performance was compared with those of the standard diagnostic tools (PSA, PSA density, and the PI-RADS score) and that of a statistical model based on logistic regression (LR).

## 2. Materials and Methods

### 2.1. Study Population

To obtain the cohort for identifying the dataset, we used the FB database of San Luigi Hospital. We extracted all the patients who underwent FB from March 2014 to December 2019, while patients who underwent FB from August 2020 to April 2021 were included as a validation set. 

Patients who were suspected of PCa (high PSA and/or positive DRE [cT2] and/or a family history of PCa), with at least one target area at mpMRI, were included. 

For each patient, we collected information about patient age, serum PSA, PSA density (according to MRI-measured prostate volume), DRE, prostate and lesion volume, lesion localization, number of suspicious lesions at mp-MRI, PI-RADS score, Gleason score (GS), number of total and positive cores, total and maximum cancer core length (CCL), and maximum CCL. The class associated with each patient was assigned according to the outcome of the FB. The detection rate (DR) was defined as the ratio between the number of PCa cases or csPCA cases identified via FB and the total number of patients who were included.

The study was performed in conformity with good clinical practice guidelines and was approved by our local ethical committee (Notifica 9/2022).

### 2.2. mp-MRI and Biopsy Technique

Suspicious lesions, detected using multiparametric MRI as reported in the ESUR guidelines, were classified using a PI-RADS score V.1 and V.2 [10].

All the operators who performed FBs had already walked their biopsy learning curve [11].

BioJet fusion system software (D&K Technologies, Barum, Germany), as previously described [12], was used to perform all the biopsies. Posterior lesions were sampled using an 18-degree transrectal needle guide, while lesions located in the transitional zone and lateral lesions were approached using a 42-degree needle. Central or anterior lesions were sampled using a transperineal approach. Depending on a lesion’s dimension (i.e., <8 mm or >8 mm), a total of four or six samples were obtained in a medio-lateral manner, according to our previously published experience [13]. In this specific study, even if a patient had undergone a concomitant standard biopsy, only the FB samples were considered.

### 2.3. Histopathological Evaluation

A single expert uropathologist (EB) evaluated all the samples, following the standards of reporting for MRI-targeted biopsy studies (START) criteria [14]. START criteria for target biopsy were used to classify clinically significant PCa (CsPCa) (i.e., biopsy GS > 7 or maximum CCL > 5 mm) [14]. For this specific study, we evaluated the index lesion only as described by Russo et al. [15].

### 2.4. ML Methods

The proposed system was based on the following four ML methods.

A fuzzy inference system (FIS) is a classifier, based on FL, using a set of fuzzy input and output variables and a list of rules for assigning the class to a given element. An FIS includes four main components: a fuzzifier, a rule base, an inference engine, and a defuzzifier. A fuzzifier converts the crisp (numeric or categorical) inputs to fuzzy sets, using membership functions. A rule base contains a number of “IF…THEN” rules that describe the relationship between the inputs and the outputs. An inference engine applies the rules to the fuzzy inputs and produces fuzzy outputs. A defuzzifier converts the fuzzy outputs to crisp (numeric) values.

The support vector machine (SVM) is a classifier belonging to the ML field that projects input data that belong to two different classes (usually nonlinearly separable) in a new higher dimensional space, in which the classes are separable by means of a hyperplane. This mapping is obtained using a function (linear or nonlinear) called *kernel function*.

The k-nearest neighbors (KNN) is a classification algorithm in which a new element is assigned to the class that is most common among its k-nearest elements in the training set, identified using a given distance metric.

Self-organizing maps (SOMs) are unsupervised artificial neural networks that are able to cluster elements in homogeneous groups. An SOM is made of a single layer of weighted and connected neurons that compete among themselves: when an input element is presented to the network, the closest neuron is found, based on its weights. This neuron is called the winning neuron. Then, the weights of the winning and the neighboring neurons are updated to make them more similar to the input element, using the following equation:

$$w_{i}^{new}=w_{i}^{old}+\varphi (n)\;▪\;\eta \;▪\;\left(x-w_{i}^{old}\right)$$

where $w_{i}^{new}$ and $w_{i}^{old}\;$ are the new and the old neuron weights, respectively; x is the input element; φ(n) is a neighborhood function; and η is the learning rate. 

### 2.5. Classification System Development

The first part of the system consists of an FIS based on the following six prebiopsy variables: PSA density, digital rectal examination (DRE), previous FBs, the number of suspicious lesions at mp-MRI, lesion location, and the PI-RADS score. This set of variables was selected from all available prebiopsy variables, based on an exhaustive search: each possible variable subset was used as input for a KNN classifier, with k = 33 and the Gower distance as the distance metric [16]. The selected best variable subset was the one that allowed the highest classification performance to be reached.

Each fuzzy input variable was modeled with a set of trapezoidal or triangular membership functions (MFs), according to the variable. The FIS output variable was associated to the patient class and modeled with two triangular MFs (negative and positive).

The set of the “IF…THEN” rules used by the FIS was obtained with an automated procedure, based on clustering and association mining through the frequent pattern (FP)-growth algorithm, which allows frequent pattern generation based on a compressed representation of the elements in a dataset called the FP tree [17]. The procedure consisted of four steps:

1. For each class, a 4-by-4 SOM was used to cluster patients into subgroups with similar characteristics. In particular, we used a linear learning function that was equal to 1 at the winner neuron, 0.5 for neurons at distance 1 from the winner neuron, and 0 otherwise; the learning rate η was set to 0.4.
2. Each input variable was transformed into a binary variable by dividing it into intervals and associating each couple variable-interval to a new binary variable: for each original value that the variable assumed for a given patient, a “1” was set in the corresponding couple variable-interval. This step was introduced because the FP-growth algorithm that is applied to the next step works only on binary data.
3. The FP-growth algorithm was applied to the binary matrix associated with each cluster and returned a list of “IF … THEN” rules [18].
4. The number of patients matching each rule was counted for the two classes separately and the rule predominance was calculated as the ratio between the number of elements belonging to the two classes: only rules that showed a predominance of elements belonging to one class were input into the FIS.

This procedure allowed the automatic extraction of a total of 29 rules: 21 rules for the classification in the negative class and 8 rules for the positive class. The resulting FIS was used to classify the patients in the training set and the patients in the validation set: the value of a patient’s prebiopsy variables was entered in the FIS and, if one or more rules were matched, one of the two classes (positive or negative) was assigned; otherwise, the patient was labeled as not classified (NC). 

An SVM was employed in cascade to the FIS, in order to manage the patients who were labeled as NC by the FC. In particular, an SVM was constructed using the entire training set and the same six input variables as those of the FIS. A linear kernel Φ was used for the mapping, according to the following equation:

$$\Phi \left(x_{i},x_{j}\right)=x_{i}^{T}x_{j}$$

Only those patients who were NC as a result of the previous classification step were input into the SVM, which assigned them one class—positive or negative. Thus, by combining FIS and SVM, each patient in the training set and in the validation set was classified as positive or negative.

**Figure 1:**
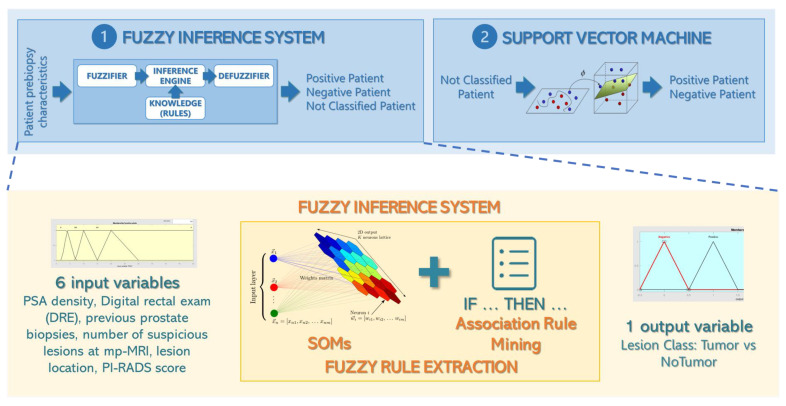
Workflow diagram of the proposed system: the first part of the system consists of an FIS based on six prebiopsy variables that assigns the patient to one of the two classes (positive or negative) or labels him as not classified. In the latter case, an SVM was employed in cascade to the FIS in order to classify such patients as positive or negative.

A simple workflow of the proposed system is shown in Figure 1.

### 2.6. Performance Evaluation

The system based on FL + SVM was compared with the following tools:

- Logistic regression (LR), a statistics-based model that captures the relationship between the categorical dependent variable (in this case, the patient class) and one or more of the independent variables (in this case, we used the same six prebiopsy variables used as input for our PPM);
- The standard diagnostic tools, i.e., PSA, PSA density (PSAdens), and the PI-RADS score.

All models were constructed using only patients in the training set and were evaluated on both the training and validation sets. The results were analyzed in terms of: the number of true positives (TPs), the number of true negatives (TNs), false positives (FPs), false negatives (FNs), negative predictive values (NPV = TN/(TN + FN)), positive predictive values (PPV = TP/(TP + FP)), specificity (spec = TN/(TN + FP)), sensitivity (sens = TP/(TP + FN)), and accuracy (acc = (TP + TN)/(TP + TN + FP + FN)). Then, the ROC curve for each model was calculated on the training set and the optimal threshold obtained from this curve was used for discriminating between the two classes [19]. Moreover, model discrimination was assessed using the area under the ROC curve (AUC) and the calculation of net benefit [20] with the Peirce formula [21]. Decision curve analysis (DCA) was implemented in order to show, graphically, the net benefit of the model. The free macro %DCA of Daniel Sjoberg for SAS ^®^ Statistics Software v 9.4 was used.

## 3. Results

### 3.1. Baseline Characteristics of Training and Validation Sets

A total of 1448 patients were included in the training set: 824 patients had positive FBs and 624 had negative FBs. One hundred and eighty-one patients were included in the validation set (119 with positive FBs and 62 with negative FBs) (Table 1).

In both sets, statistically significant differences were found between positive and negative FB patients in terms of age, PSA density, number of positive DREs, prostate and lesion volume, and PI-RADS score distribution (Table S1).

In the training and validation groups, the overall DRs were 56.9% and 65.2% (*p* = 0.04), respectively, while the DRs for csPCa were 50.4% and 56.9% (*p* = 0.11), respectively. The stratification of the DRs according to the different PI-RADS scores and ISUP grades is shown in Tables S2 and S3, respectively.

### 3.2. Evaluation of Models’ Performances in Training Set

Using only the FIS model, we were able to classify 927 patients who belonged to the training set (64.1%). The remaining 521 patients, for whom no matching rule was found, were input into the SVM, which assigned them one by one into the two classes. This cascade system allowed the classification accuracy to be improved from 48.0%, using the FIS alone, to 68.8%, when the combination of FIS and SVM was employed. 

**Figure 2:**
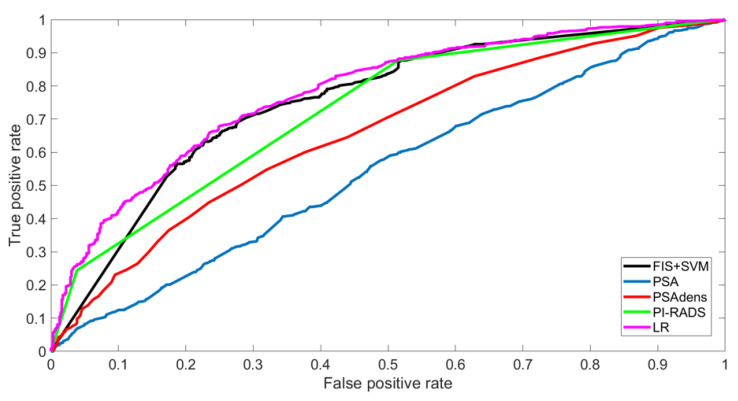
ROC curves obtained for the five models on the training set.

A comparison of the results for the training set, obtained with the proposed system and with the other four models, is reported in the upper part of Table 2. The models’ discrimination calculation showed that the AUC of the proposed FIS + SVM model was comparable to that of the LR model (0.75, CI95% 0.73–0.78 vs. 0.78 CI95% 0.76–0.80) but outperformed the other diagnostic tools (Figure 2). The optimal thresholds obtained from the ROCs were as follows: 0.5 for FIS + SVM, 0.54 for LR, 3.45 for PSA, 0.10 for PSAdens, and 4 for PIRADS. Values equal to or greater than the thresholds were assigned to the positive class.

As shown in the results, the FIS + SVM model returned the best performance, in terms of NPV, on the training set (78.5%). Moreover, the FIS + SVM model also outperformed the LR in terms of specificity (92.1% vs. 83%).

**Figure 3:**
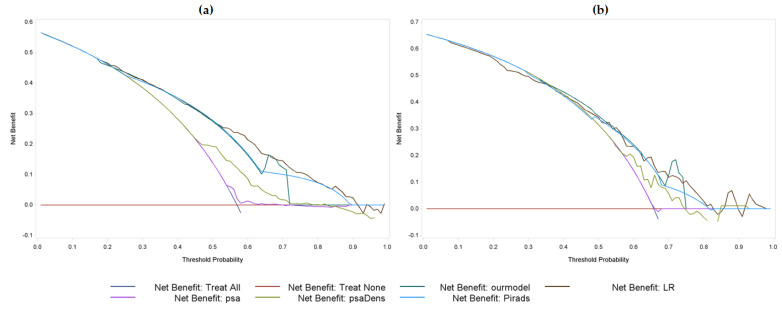
DCA for the training set (**a**) and the validation set (**b**) obtained with the five models.

The DCA for the training set is shown in Figure 3a.; The net benefit of each model is reported in the last column of Table 2. The FIS + SVM model reached a net benefit very close to the highest value associated with the PSA (0.49 vs. 0.50).

### 3.3. Evaluation of Models’ Performances in Validation Set

Considering the validation set, the FIS model alone was able to classify 127 patients (85.8%). The remaining 21 not-classified patients were assigned to one of the two classes using the SVM. In terms of classification accuracy, the combination of FIS and SVM produced an increase in accuracy from 62.1% (using only the FIS) to 69.1%. 

The results of the five models are reported in the lower part of Table 2. As shown in the table, our model outperformed the other models in terms of NPV (60.7%), sensitivity (90.8%), and accuracy (69.1%). The optimal performance in terms of the DCA were reported in Figure 3b. Finally, analyzing the 11 FN patients returned by our PPM (i.e., evaluated as negative by the system prior to a positive biopsy), the distribution of ISUP scores was as follows: 54.5% (6/11) with ISUP 1, 36.4% (4/11) with ISUP 2, 9.1% (1/11) with ISUP 3, 0% (0/11) with ISUP 4, and 0% (0/11) with ISUP 5.

## 4. Discussion

In this study, we developed and validated a PPM that was able to identify the ideal candidate for FB, based on the combination of FIS and SVM. This combination was compared with four other models (PSA, PSAD, PIRADS, and LR) in terms of the performances reached on the training and validation sets. 

The performances were good for the PCa probability model, with an AUC equal to 0.75 and an NPV of 78.5% in the training sets. Then, the capability of our system to identify PCa based on prebiopsy characteristics was tested in a prospectively enrolled cohort showing a sensitivity of 90.8% and an accuracy of 69.1%. As shown in Table 1, Tables S1 and S2, we noticed that the training and validation cohorts had different baseline characteristics and biopsy findings: however, notwithstanding these differences, our PPM was able to correctly classify the patients in 68.8% and 69.0% of the cases, respectively, overcoming this confounding bias.

In the present study, we established that PSA density, DRE, previous FBs, number of suspicious lesions at mp-MRI, lesion location, and the PI-RADS score had an impact on PCa probability. This set of variables was selected according to our previously published experience [9] using the KNN classifier and, then, the best subset was selected as the one that allowed the highest classification performance to be reached.

In some recent works, PSAdens was shown to be the most powerful risk predictor, compared to the other variables. In addition, the combination of PSAdens and MRI was evaluated, demonstrating good guidance in biopsy decision-making, allowing the avoidance of unnecessary biopsy testing, without harm. According to a meta-analysis of >3000 biopsy-naïve men, a risk-related data table of csPCa was established, evaluating both the PI-RADS scores and PSAdens, demonstrating that the likelihood of having an ISUP grade > 2 tumor in biopsy-naïve men with a PI-RADS score of 1 or 2 and a PSAD below 0.10 was 3–4%; i.e., <5% [22,23,24]. 

On the other hand, a suspected DRE is included in most risk calculators, despite its suboptimal diagnostic role due to its low PPV—estimated as <40% as demonstrated by a recent meta-analysis [24]—and by the fact that DRE is strictly operator-dependent.

Similar to our experience, Chiu et al. [25] used PSA, DRE, prostate volume, and TRUS findings as biopsy parameters for their ML model, while Suh et al. [26] established that PSA level, age, TZ volume, total volume, testosterone level, HypoE on TRUS Bx_N, and fPSA level had an impact on PCa probability.

Furthermore, trying to better explain how our model was developed, the following aspects should be mentioned. In particular, the AUC on the training set was employed to obtain the best threshold for the classification of positive and negative patients. This procedure ensured the maximization of the true positive rate, while minimizing the false positive rate for each model, although it implied the use of thresholds that were different from those generally used in clinical practice for the three standard diagnostic tools, i.e., PSA < 4 ng/dL, PSA density < 0.15, and a PI-RADS score < 3. 

We were unable to assess the calibration between observed probabilities and predicted probability by means of calibration plots, as the proposed model (FIS + SVM) was obtained as a cascade of two classifiers and did not return a probability as an output. 

Focusing on the validation set, the proposed FIS + SVM reached the best results in terms of accuracy (69.1%), sensitivity (90.8%), and NPV (60.7%). In particular, the last parameter was crucial in this application, because our aim was to develop a model with two simultaneous characteristics: (1) a high value of TNs to reduce the number of useless biopsies, and (2) a low number of FNs to reduce the number of positive patients who were not correctly recognized by the system. None of the other models achieved similar results in terms of NPV on the validation set. The model based on LR was able to correctly recognize the highest number of negative patients (TN = 28) but, at the same time, the number of mistakes for the positive patients was the highest among all models (FN = 26). The PSA reached the same sensitivity as FIS + SVM (90.8%), which was, however, associated with the lowest specificity value (6.5%). Neither the PSA density nor the PI-RADS score reached satisfactory results on the validation set, when considered alone.

At the sensitivity of 90.8% for PCa, FIS + SVM model achieved the best NPV of 60.7%, and 15.46% (28/181) of prostate biopsies could have been avoided in the validation set. Furthermore, we observed that the absence of FN was recorded in ISUP 3, 4, and 5, and only one patient was erroneously classified in the ISUP 2 group, proving again the optimal capability of our PPM to correctly identify patients with PCa, especially in cases of csPCa

**Figure 4:**
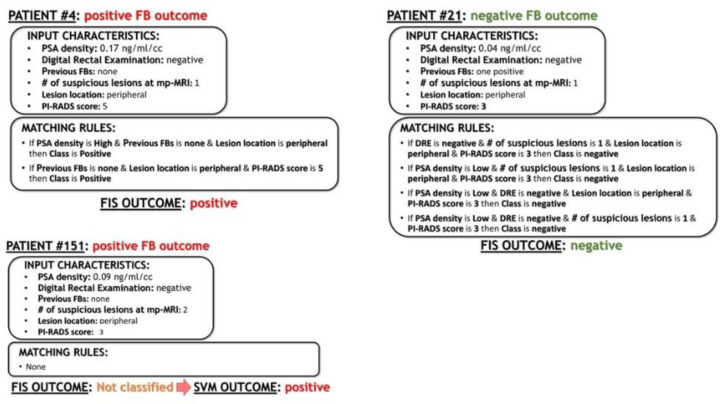
Three examples of patients belonging to the validation set classified using our FIS + SVM model. For each patient, the list of the “IF…THEN” rules matching with the patient’s input characteristics is reported, together with the FB outcome and the outcome of our system.

Then, regarding other AI and ML tools, which are mainly black boxes from the perspectives of explainability and interpretability, our system, which is based on a fuzzy inference system, supports users in the interpretation of results. In recent years, growing attention has been paid to this aspect, especially when ML and AI tools are employed in critical domains such as healthcare [27]. In fact, understanding the outcome suggested by this kind of system is of crucial importance for decision makers (in this case clinicians) in order to trust the system and to make informed decisions. As the FIS is a rules-based system, and rules are commonly used by humans in their daily lives, our proposed method facilitates an easy understanding of the basis upon which a class is assigned to a certain patient, including classifications by users who do not have a technical background. In particular, when a class is assigned to a certain patient, it is possible to know, exactly, the subset of the “IF-THEN” rules that match the patient’s input characteristics (some examples are reported in Figure 4). In this case, the resulting interpretation is improved by two factors that characterize our FIS: the reduced number of input variables (there are only six), all with a well-defined clinical meaning, and the limited number of obtained rules (there are only 29). Such evaluations of results is not feasible via most standard statistical methods, such as LR, or via other ML tools, such as artificial neural networks, for which only prediction results can be obtained, without any support for the interpretation of the decision.

Finally, we emphasize that this is the first study to develop a specific AI-based risk calculator based only on FB samples, following the direction of precision medicine [28] and the current trends in prostate cancer diagnosis [29,30] (Figure 4).

The major limitation of the present study is that it was retrospectively designed and was carried out in a single center; thus, external validation is required. Another possible limitation is the absence of radiological features directly extracted from MRI images. In the future, the implementation of our algorithm with radiomics features can help to further improve its performance. Notwithstanding these limitations, our work demonstrates relevant advantages of the machine-learning model, in accordance with the common data science pipeline, with easy management and upgrading. Most importantly, we successfully developed and validated a method, based on fuzzy logic, that facilitates an explanation to physicians of the result of the classification of each single patient, in terms of rules and clinical variables.

## 5. Conclusions

We created and validated a tool using FIS + SVM for the definition of the risk of PCa before the execution of a target fusion biopsy. Our models demonstrated very promising performances regarding the prediction of PCa, avoiding the execution of pointless biopsies in 15% of the cases. Moreover, the relevance of each parameter could be evaluated using the “IF…THEN” rules. Thanks to this AI-based model, patient selection and counseling might be further improved, following the concept of the “precision surgery era”.

## Figures and Tables

**Table 1:** Demographic characteristics of the study population.

	Overall (1629 pts)	Training Set (1448 pts)	Validation Set (181 pts)	*p*-Value
Age, years; mean (SD)	69 (7.9)	69 (8.0)	68 (7.8)	0.11
PSA, ng/dL; mean (SD)	8.2 (6.8)	8.4 (7.5)	7.6 (4.7)	0.16
PSA density, ng/mL^2^; mean (SD)	0.16 (0.15)	0.17 (0.17)	0.13 (0.13)	0.0025
Positive DRE, number (%)	220 (13.5)	177 (12)	43 (23.7)	<0.001
Prostate volume, CC; mean (SD)	54.2 (26.6)	55.8 (27.8)	50.8 (25.5)	0.02
Lesion volume, CC; mean (SD)	0.96 (1.56)	0.98 (1.91)	0.88 (1.24)	0.49
Pirads score 3 4 5				0.16
	449 (27.6)	403 (27.9)	46 (25.4)	
	917 (56.3)	820 (56.6)	97 (53.6)	
	263 (16.1)	225 (15.5)	38 (21.0)	
Lesion location, number (%) Peripheral Transitional/anterior				0.65
	1395 (85.6)	1238 (85.5)	157 (86.7)	
	234 (14.4)	210 (14.5)	24 (13.3)	
Biopsy approach, number (%) Transrectal 18° needle guide biopsy, number (%) Transrectal 42° needle guide biopsy, number (%) Transperineal, number (%)				<0.001
	470 (28.8)	413 (29)	57 (31.5)	
	965 (59.2)	841 (58)	124 (68.5)	
	194 (12)	194 (13)	0 (0)	
Overall detection rate of PCa, number (%)	942 (57.8)	824 (56.9)	118 (65.2)	0.04
Overall detection rate of csPCa, number (%)	833 (51.1)	730 (50.4)	103 (56.9)	0.11

FB: fusion biopsy; SD: standard deviation, PSA: prostate specific antigen; DRE: digital rectal examination; CC: cubic centimeters; PCa: prostate cancer; csPCa: clinically significant PCa.

**Table 2:** Results of the five models for the training and the validation sets. Values in bold represent the best performances for each set.

		AUC (95%CI)	TP	TN	FP	FN	NPV	PPV	Spec	Sens	Acc	Net Benefit
**TRAINING SET**	FIS + SVM	0.75 (0.73–0.78)	759	238	385	65	78.5%	66.3%	38.2%	92.1%	68.9%	0.49
	LR	0.78 (0.76–0.80)	684	360	263	140	72.0%	72.2%	57.8%	83%	72.1%	0.45
	PSA	0.55 (0.51–0.57)	792	52	571	32	61.9%	58.1%	8.3%	96.1%	58.3%	0.50
	PSAdens	0.66 (0.63–0.68)	683	232	391	141	62.2%	63.6%	37.2%	82.9%	63.2%	0.44
	PI-RADS	0.73 (0.70–0.75)	723	302	321	101	74.9%	69.3%	48.5%	87.7%	70.8%	0.48
**VALIDATION SET**	FIS + SVM	-	108	17	45	11	60.7%	70.6%	27.4%	90.8%	69.1%	-
	LR	-	93	28	34	26	51.9%	73.2%	45.2%	78.2%	66.9%	-
	PSA	-	108	4	58	11	26.7%	65.1%	6.5%	90.8%	61.9%	-
	PSAdens	-	96	19	43	23	45.2%	69.1%	30.6%	80.7%	63.5%	-
	PI-RADS	-	98	25	37	21	54.3%	72.6%	40.3%	82.4%	68.0%	-

FIS: fuzzy inference system, SVM: support vector machine, LR: logistic regression, PSA: prostatic specific antigen; PSAdens: PSA density: PI-RADS: prostate imaging-reporting and data system.

## Data Availability

Not applicable.

## Supplementary Materials

The following supporting information can be downloaded at: https://www.mdpi.com/article/10.3390/jcm12134358/s1, Table S1: Characteristics of the mp-MRI lesions identified; Table S2: Cancer detection rates of positive fusion biopsies; Table S3: Histopathologic characteristics of the study population.
