# Supplementary material for: Machine-Learning-Based Tool to Predict Target Prostate Biopsy Outcomes: An Internal Validation Study
Source: J Clin Med. 2023 Jun 28;12(13):4358. doi: 10.3390/jcm12134358 (PMC10342762; doi:10.3390/jcm12134358)
Supplement: Supplementary file 1 [file jcm-12-04358-s001.zip › jcm-2465204-SI.pdf]

mp-MRI characteristics	Training set with positive FB	Training set with negative FB	p-value	Validation set with positive FB	Validation set with negative FB	p-value
**Number of lesions**	824	624		119	62	
**Right lesion, number (%)**	440 (53,4)	322 (51,6)	0.53	54 (45,4)	21 (33,9)	0.18
**Left lesion, number (%)**	378 (45,9)	308 (49,4)	0.20	56 (47)	32 (51,6)	0.66
**Apical lesion, number (%)**	339 (41,2)	230 (36,9)	0.10	20 (16,8)	11 (17,7)	0.95
**Equatorial lesion, number (%)**	282 (34,2)	224 (35,9)	0.53	62 (52,1)	30 (48,4)	0.75
**Basal lesion, number (%)**	203 (24,6)	170 (27,2)	0.28	29 (24,4)	17 (27,4)	0.79
**Posteromedial lesion, number (%)**	257 (31,2)	224 (35,9)	0.06	2 (1,7)	2 (3,2)	0.90
**Posterolateral lesion, number (%)**	444 (53,9)	311 (49,8)	0.13	82 (68,9)	38 (61,3)	0.38
**Transitional zone lesion, number (%)**	123 (14,9)	89 (14,3)	0.80	10 (8,4)	12 (19,4)	0.05
**Pirads 3, number (%)**	101 (12,3)	302 (48,4)	<0.001	20 (16,8)	25 (40,3)	0.001
**Pirads 4, number (%)**	522 (63,3)	298 (47,8)	< 0.001	67 (56,3)	30 (48,4)	0.39
**Pirads 5, number (%)**	201 (24,4)	24 (3,8)	< 0.001	31 (26)	7 (11,3)	0.03

Table S1. Characteristics of the mp-MRI lesions identified.

	Overall positive FB	Positive FB of training set	Positive FB of validation set	p-value
**Overall detection rate of PCa, number (%)**	943 (57,9)	824 (56,9)	119 (65,2)	0.04
**Overall detection rate of csPCa, number (%)**	834 (51,2)	730 (50,4)	104 (56.9)	0.011
**Ratio of overall detection of csPCa/PCa, %**	88,4	88,6	87,4	0,92
**Pirads 3 lesions**				
**Overall detection rate of PCa, number (%)**	121 (27)	101 (25,1)	20 (44,4)	0.008
**Overall detection rate of csPCa, number (%)**	84 (18,8)	73 (18,1)	11 (24,4)	0.04
**Ratio of overall detection of csPCa/PCa, %**	69,4	72,3	55	0,49
**Pirads 4 lesions**				
**Overall detection rate of PCa, number (%)**	589 (64,2)	522 (63,7)	67 (69,1)	0.34
**Overall detection rate of csPCa, number (%)**	522 (56,9)	460 (56,1)	62 (63,9)	0.17
**Ratio of overall detection of csPCa/PCa, %**	88,6	88,1	95,5	0,79
**Pirads 5 lesions**				
**Overall detection rate of PCa, number (%)**	231 (87,8)	201 (89,3)	31 (81,6)	0.27
**Overall detection rate of csPCa, number (%)**	227 (86,3)	197 (87,6)	30 (78,9)	0.45
**Ratio of overall detection of csPCa/PCa, %**	98,3	98,0	96,8	0,96

FB: fusion biopsy; PCa = prostate cancer; csPCa = clinical significant prostate cancer

Table S2. Cancer detection rates of positive fusion biopsies.

	Overall positive FB	Positive FB of training set	Positive FB of validation set	p-value
**PCa, number (%)**	943 (57,8)	824 (56,9)	119 (65,7)	0.04
**Biopsy GS, number (%)**				
• **6**	109 (11,6)	94 (11,4)	15 (12,6)	0,48
• **7**	656 (69,6)	580 (70,4)	76 (63,9)	
• **8**	119 (12,6)	101 (12,3)	18 (15,1)	
• **>8**	59 (6,3)	49 (5,9)	10 (8,4)	
**Total CCL, mm; mean (SD)**	19,8 (17)	18,2 (16,5)	21,4 (17,9)	0.050
**Maximum CCL, mm; mean (SD)**	7,4 (4,5)	7,0 (4,5)	7,7 (4,4)	0.11
**Maximum CCI rate, mean (SD)**	52,9 (29)	52,9 (27,3)	53 (32,8)	0.97

FB: fusion biopsy; PCa = prostate cancer; CCI = cancer core invasion; CCL = cancer core length; GS = Gleason score; SD: standard deviation

Table S3. Histopathologic characteristics of the study population.
